# The cardiosplenic axis: the prognostic role of the spleen in heart failure

**DOI:** 10.1007/s10741-022-10248-4

**Published:** 2022-05-18

**Authors:** Hiroaki Hiraiwa, Takahiro Okumura, Toyoaki Murohara

**Affiliations:** Department of Cardiology, Nagoya University Graduate School of Medicine, 65 Tsurumai-cho, Showa-ku, Nagoya, 466-8550 Japan

**Keywords:** Cardiosplenic axis, Heart failure, Spleen, Prognosis, Hemodynamics, Immunity

## Abstract

Despite the number of available methods to predict prognosis in patients with heart failure, prognosis remains poor, likely because of marked patient heterogeneity and varied heart failure etiologies. Thus, identification of novel prognostic indicators to stratify risk in patients with heart failure is of paramount importance. The spleen is emerging as a potential novel prognostic indicator for heart failure. In this article, we provide an overview of the current prognostic tools used for heart failure. We then introduce the spleen as a potential novel prognostic indicator, before outlining the structure and function of the spleen and introducing the concept of the cardiosplenic axis. This is followed by a focused discussion on the function of the spleen in the immune response and in hemodynamics, as well as a review of what is known about the usefulness of the spleen as an indicator of heart failure. Expert insight into the most effective spleen-related measurement indices for the prognostication of patients with heart failure is provided, and suggestions on how these could be measured in clinical practice are considered. In future, studies in humans will be required to draw definitive links between specific splenic measurements and different heart failure manifestations, as well as to determine whether splenic prognostic measurements differ between heart failure classes and etiologies. These contributions will provide a step forward in our understanding of the usefulness of the spleen as a prognostic predictor in heart failure.

## Introduction

Cardiovascular diseases contribute to an estimated 17.9 million deaths each year [1]. Despite advances in heart failure management and treatment, its prevalence was 64.63 million cases worldwide in 2020, and this figure continues to increase owing to population aging [2]. An estimated 1–3% of adults are living with heart failure [3], and prognosis is poor, with 5- and 10-year mortality rates of 50% and 90%, respectively [4].

There is substantial heterogeneity in the clinical progression of patients with heart failure because of marked patient heterogeneity [5–8] and varied heart failure etiologies, and a deeper understanding of this heterogeneity is needed to improve patient care. This limited understanding impedes the development of effective strategies to address heart failure as a public health concern [8]. The complexity of heart failure dictates that personalized medicine is fundamental for treatment and management. Precision medicine is also key, and improving the accuracy of risk prediction requires the incorporation of several clinical variables that are routinely assessed in clinical practice [9]. As such, prognostic models for heart failure that incorporate several clinical variables tend to be more useful than assessment of individual clinical parameters [9].

### Heart failure prognostic prediction

There are a number of traditional and established predictors of prognosis in heart failure; however, many have their shortcomings. Raphael et al. [10] emphasize high inter-operator variability in the methods used to assess New York Heart Association (NYHA) functional class in clinical practice. Another study has shown comparable mortality rates between patients with NYHA functional class I or II and those with NYHA functional class III or IV [11]. In terms of prior heart failure hospitalization, Blumer et al. [12] showed that prior heart failure hospitalization is not associated with 180-day mortality and suggest that clinical variables may be a more direct means of predicting prognosis in patients with heart failure.

Other factors have also been investigated as prognostic determinants in patients with heart failure. A lower resting cardiac power output has been demonstrated as a prognostic factor in patients with advanced disease after adjusting for certain variables, including age, sex, left ventricular ejection fraction (LVEF), and mean arterial pressure [13]. Moreover, a low cardiac power index has been shown to predict a high cardiac event rate in patients with NYHA functional class II/III heart failure [14]. Right heart catheterization (RHC) is also used to assess hemodynamics in patients with heart failure; however, owing to its invasive nature, RHC is associated with an increased risk of complications [15]. Moreover, in patients with heart failure undergoing implantable cardioverter-defibrillator implantation, an increase in heart rate is strongly associated with poor outcomes [16, 17]. Recently, the value of the plasma amino acid profile as a novel prognostic marker for heart failure has been investigated, including the leucine/phenylalanine ratio, which was an independent predictor of future cardiac events in patients with heart failure [18, 19]. Despite the number of prognostic prediction methods to stratify risk and the number of treatment options in patients with heart failure, prognosis remains poor, which may be due to the substantial inter-patient heterogeneity, variation in heart failure etiologies, and the focus on symptom control with existing therapies. Thus, both treatment and prognostic prediction require improvement [20]. Novel prognostic indicators that can be incorporated into effective prognostic models are of paramount importance to effectively stratify risk and improve outcomes for patients.

Although the spleen has received limited research attention thus far, it is emerging as a potential prognostic indicator in patients with heart failure based on new evidence that highlights crosstalk between the heart and spleen, termed the “cardiosplenic axis” [18, 21–23]. Although the cardiosplenic axis has been widely demonstrated in animals, only a few studies have demonstrated its presence in humans [21–23]. Therefore, little is known about the potential of the cardiosplenic axis for clinical application [24].

This review will describe the structure and function of the spleen and discuss the concept of the cardiosplenic axis. This will be followed by a focused discussion on the function of the spleen in the immune response and in hemodynamics, as well as a review of what is known about the usefulness of the spleen as an indicator of cardiovascular disease and heart failure. Expert insight into the most effective spleen-related measurement indices for the prognostication of patients with heart failure will be provided, and suggestions on how these could be measured in clinical practice will be considered. In this review, we aim to collate current knowledge to provide insight into how this area of research could be carried forward for potential application in the clinic.

## Biology of the spleen

### The structure and function of the spleen

The spleen performs a number of functions that are facilitated by its structural components. The spleen varies in size between individuals, but it is typically ~ 10 × 6 × 3 cm^3^, weighs ~ 120 g, and is highly vascularized, storing 20–30% of total blood volume [21]. Larger-than normal spleen sizes have been reported in “Sea Nomads” (or Bajau people) who engage in breath-hold diving and in elite endurance athletes [25, 26]. Although splenic contraction is a part of the human diving response, it is thought that those who engage in breath-hold diving have physiologically and genetically adapted to the hypoxic environment, which has led to the development of a larger-than normal sized spleen. The larger splenic volume in elite endurance athletes is possibly functional and helps to cope with oxygen demands and deficits. Structurally, the spleen can be divided into two main regions, which are distinguished as white pulp and red pulp. The latter comprises the majority of splenic tissue. In the red pulp, pathogens, cellular debris, and old erythrocytes are efficiently removed from the circulation, and iron is recycled by an abundance of macrophages. The white pulp is the primary immunological region of the spleen in rodents and humans. It mostly consists of lymphoid cells in a highly organized compartment in which adaptive immune responses are initiated.

Functionally, the red pulp plays a role in immunity that is distinct from that of the white pulp [27]. Leukocytes residing in the red pulp, including neutrophils, monocytes, dendritic cells, and macrophages [28, 29], exit the spleen mostly through splenic veins in the red pulp [30]. The white pulp functions as a secondary lymphoid organ of the circulatory system and contains T and B cell zones in which antigen-specific immune responses are generated. The spleen regulates innate and adaptive immunity, contributing to immune responses that can either protect the host or contribute to disease, and can effectively clear pathogens from the blood [31].

### The cardiosplenic axis

The relationship between the spleen and heart was first identified by Rein et al. in 1949 [32], who showed that electrical stimulation of splenic nerves restored ventricular function in dogs. Rein and colleagues also showed that splenectomy worsened myocardial contractile failure [33]. On the basis of these observations, the authors speculated a role for the spleen in protecting and maintaining cardiac function. Attention to this topic subsequently declined until 2009 when Swirski et al. investigated the role of the spleen as a reservoir for inflammatory macrophages and monocytes [34]. The authors showed that following myocardial infarction, splenic activation by increased angiotensin II mobilized splenic monocytes in mice, which infiltrated the myocardium.

Since these initial observations, the spleen has been further shown to influence the function of the heart, providing further evidence of the cardiosplenic axis. A key observation is that splenic contraction and volume reduction counteract tissue hypoperfusion. Splenic contraction is mediated by sympathetic control and is intended to compensate for systemic oxygen demand. This is associated with various states, such as hypovolemic shock [35, 36], sepsis [37, 38], hypoxia, trauma [39], and exercise [40, 41], supporting a hemodynamic link [21] between the spleen and the heart.

Acute myocardial infarction is one example of a cardiovascular event that increases sympathetic nervous system activity, which mobilizes progenitor cells from the bone marrow, in turn boosting monocyte production in the spleen [42]. Beta-adrenoreceptors are involved in leukocyte mobilization from the spleen [42]. Moreover, beta-adrenoreceptor activation increases interleukin-10 (IL-10) mRNA expression in the spleen and reduces infarct size in mice, which is abrogated by splenectomy; thus, IL-10 produced in the spleen might exert a cardioprotective effect [43, 44].

Further links between the spleen and the heart have also been noted. For example, positive inotropy has been reported in isolated cardiac preparations when exposed to splenic extracts [45]. Moreover, Emami et al. [23] have shown that the metabolic activity of the spleen markedly increases after acute coronary syndrome in humans and is associated with an increased risk of subsequent cardiovascular events. Additionally, in a mouse model of myocardial infarction with pre-existing chronic inflammation, angiotensin-converting enzyme inhibition has been shown to prevent monocyte release from the splenic reservoir [46]. All of the above evidence suggests a close link between the spleen and the heart.

## The cardiosplenic axis in heart failure

### The function of the spleen in the immune response

**Fig. 1:**
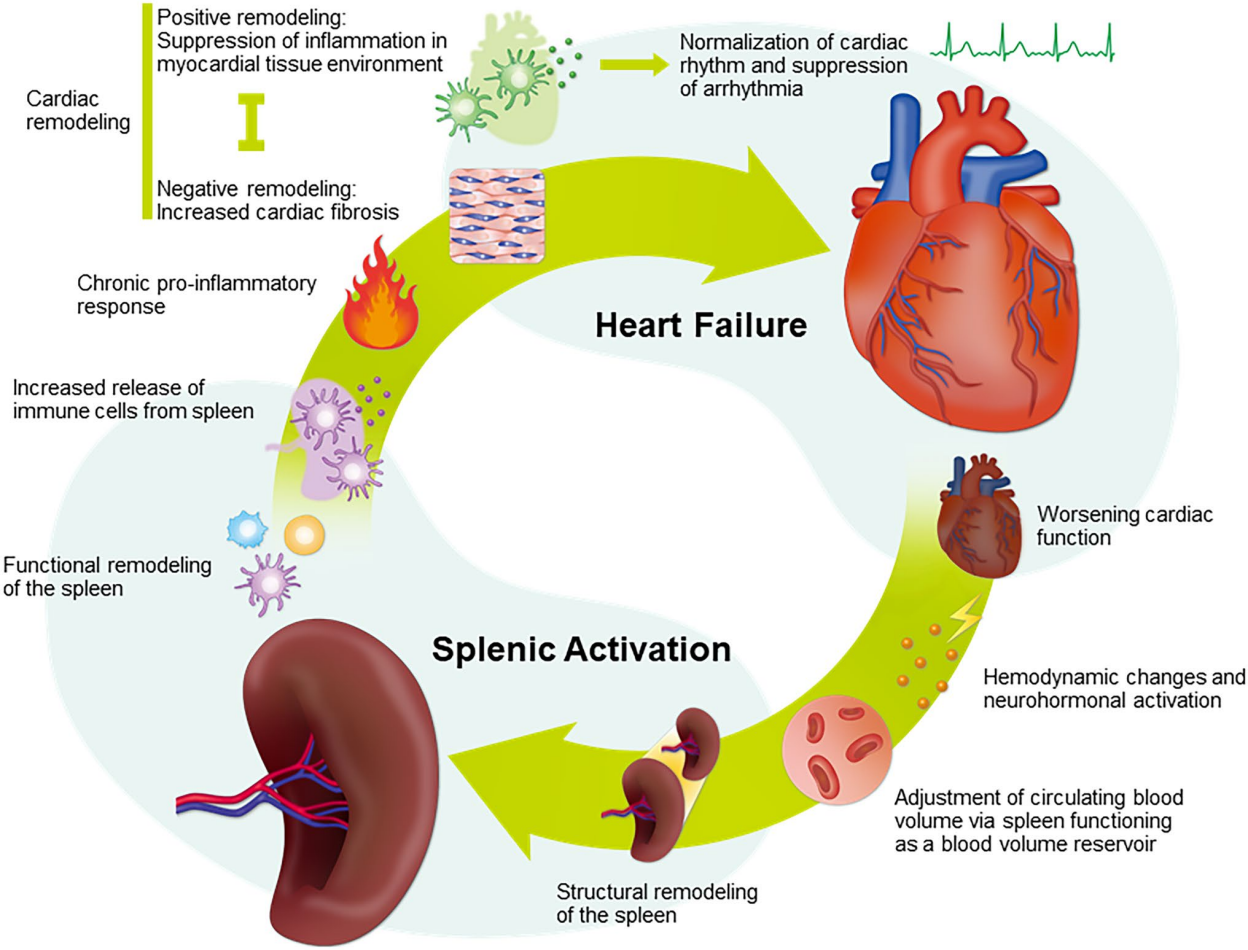
Proposed mechanisms of the cardiosplenic axis in heart failure. The cardiosplenic axis can be described as a cycle involving hemodynamic and immune processes. Heart failure is caused by reversible or irreversible damage to cardiac muscle. This tissue damage leads to immune cell recruitment from the circulation, triggering inflammation through cross-talk with the spleen. In terms of hemodynamic changes, the spleen holds a significant amount of the increased intravascular blood volume and regulates fluid distribution. As a result, splenomegaly and increased spleen stiffness are often observed in heart failure. This structural remodeling of the spleen is accompanied by functional remodeling, with changes in the composition of spleen-derived immune cells. Splenic immune cells, such as splenic macrophages, are released into the blood and migrate to the heart. This can lead to cardiac remodeling at the macroscopic and microscopic levels, which may be maladaptive or adaptive. The former can lead to cardiac fibrosis, while the latter is associated with suppression of inflammation in myocardial tissue via cardiac macrophage activity, which supports normalization of cardiac rhythm and suppression of arrhythmia development. These seemingly contradictory mechanisms have antagonistic effects, and their balance may affect cardiac function and prognosis. Owing to these wide-ranging changes, the spleen may be a clinical prognostic indicator in heart failure. For example, changes at the macroscopic level (spleen size and stiffness), as well as at the microscopic level (specific subsets of circulating spleen-derived immune cells), could be utilized as biomarkers to predict the prognosis of patients with heart failure

The spleen is an important reservoir for the deployment of leukocytes to tissue sites of injury and inflammation [34, 47]. In a study by Prabhu [24], mice with ischemic heart failure demonstrated expansion of proinflammatory monocytes and macrophages, as well as splenic remodeling. However, splenic macrophages are proinflammatory and promote tissue injury [24]. For instance, activation of splenic immune cells underlies the chronic inflammatory response in heart failure, and these cells induce tissue injury, leading to cardiac remodeling (Fig. 1). This process is facilitated by heightened antigen processing in the spleen [48]. Another study by Fujinami et al. [49] showed that the number of peripheral blood monocytes, which is positively correlated with maximum spleen diameter and spleen index, is related to the response to cardiac resynchronization therapy in chronic heart failure. Cardiac tissue damage, stress signals, and inflammation can activate the innate immune system, triggering the release of splenic immune cells, which can further contribute to chronic inflammation and cardiac remodeling in heart failure; thus, immune cell-mediated inflammation can play both a causative and consequent role in cardiac remodeling and heart failure [50]. Therefore, the interactions between the spleen and heart in terms of immune-mediated inflammation appear to be complex and bidirectional*.*

In mice, the cancer therapeutic doxorubicin disrupted immunometabolic pathways, triggered by persistent inflammation in the spleen and heart [51]. Specifically, doxorubicin dysregulated cyclooxygenase in the spleen and left ventricle. As a result, cyclooxygenase-mediated prostaglandin species were decreased in the left ventricle [51]. Doxorubicin causes cardiac toxicity and heart failure [52]; thus, an understanding of the immunometabolism pathways within the spleen and heart may be important for improving protection from heart failure, and this may be a key component of the cardiosplenic axis.

Like the spleen, the myocardium has its own resident macrophages; however, unlike spleen-derived macrophages, cardiac macrophages are minimally inflammatory and promote angiogenesis and tissue repair [24]. During the course of heart failure, macrophages gradually expand within the remote myocardium [53]. These immune cells are thought to phagocytose material from cardiomyocytes, such as remnants of dysfunctional mitochondria, through a process driven by cardiomyocyte autophagy during cardiac stress [54]. Cardiac macrophages also facilitate electrical conduction through the atrioventricular node. Cardiac macrophages, which reside between cardiomyocytes and in the atrioventricular node, form gap junctions with cardiomyocytes in the steady state and regulate conduction between cardiomyocytes [55]. Specifically, they have been shown to accelerate cardiomyocyte repolarization [55]. Cardiac macrophages are also involved in the normalization of cardiac rhythm and the suppression of arrhythmia development [55]. In a mouse model of heart failure, macrophages have been shown to act not only on cardiomyocytes, but also on fibroblasts via secretion of oncostatin-m [56], which directly inhibits tumor growth factor-β1-mediated activation of cardiac fibroblasts to suppress cardiac fibrosis.

It is important to note that the role of macrophages in fibrosis is not well understood owing to conflicting evidence. The conflicting observations may be due to differences in gene expression between different macrophage populations. For example, Haider et al. [57] showed that macrophages can transition to fibroblast-like cells in the heart following myocardial infarction with respect to their expression of fibroblastic markers. The triggers for such changes in gene expression are likely complex and multifactorial. For example, Nacu et al. [58] showed that macrophage phagocytosis of dead cells and debris leads to the release of pro-fibrotic mediators. Thus, it is possible that pro-fibrotic mediators may be expressed at a higher level in more advanced heart failure where tissue damage can be more severe, leading to fibrosis and maladaptive cardiac remodeling. Another study showed that sphingosine-1-phosphate (S1P) and S1P receptor expression were increased in the heart and spleen of mice. Selective activation of the S1P receptor in macrophages suppressed the inflammatory markers tumor necrosis factor-alpha and monocyte chemoattractant protein-1 (CCL2), expedited the reparative marker arginase-1, and was indicative of cardiac repair in the acute phase of heart failure. The study suggested that alterations in the S1P pathway in macrophages may contribute to pathological remodeling in heart failure [59]. There are a multitude of anti- and pro-inflammatory mediators that contribute to remodeling in the different stages (inflammatory, reparative, proliferative, and maturation), and some cell populations, such as macrophages, have two or more subsets with opposite or complementary actions, which have been discussed in detail previously [60, 61]. Hence, the balance between different macrophage populations may determine the status of remodeling and inform heart failure prognosis.

### The function of the spleen in hemodynamics

In addition to its function in immunity, the spleen also plays a role in hemodynamics. A previous study has shown that splenic size increases in patients with advanced heart failure after left ventricular assist device (LVAD) implantation [21]. Another study in patients with advanced heart failure found a significant negative correlation between splenic volume and systemic vascular resistance prior to LVAD implantation, which was absent after LVAD implantation. These observations suggest that the spleen serves as a blood volume reservoir for systemic volume regulation (Fig. 1). Ibrahim et al. [62] demonstrated splenic enlargement in congestive heart failure. As such, spleen size may change in patients with heart failure undergoing LVAD support as a response to a change in cardiac preload and systemic volume status. Whether spleen size could be useful to monitor preload and systemic volume status as indicators of heart failure prognosis remains to be clarified.

Fujinami et al. [49] observed that chronic heart failure induces splenomegaly and that spleen size could predict the response to cardiac resynchronization therapy. A previous study has shown that splenectomy in mice with heart failure results in left ventricular reverse remodeling, with a reduction in left ventricular chamber size and an improvement in LVEF [24]. This research group proposed that a pathological cardiosplenic axis develops during heart failure, which is essential for disease progression. Furthermore, Erdem et al. [63] reported on the clinical course of five children with asplenia and concomitant cardiac abnormalities, and showed that congenital asplenia was associated with ventricular and atrial septal defects. Conversely, another study has shown that splenectomy reduces inflammatory infiltration and improves cardiac geometry and function [48]. Hence, there are conflicting reports as to whether splenectomy has a positive or negative effect on cardiac function. Moreover, it is not clear what effect splenectomy has on prognosis, which may be worthy of further investigation.

A recent study has shown that splenic volume is correlated with right arterial pressure and pulmonary capillary wedge pressure [64]. Moreover, heart rate and splenic volume are independent determinants of pulsatility index, reflecting cardiac preload in patients with heart failure [64]. This study concluded that spleen measurements may help to estimate systemic volume status and to understand hemodynamics in patients with LVADs for heart failure. Another recent study has shown an association between spleen volume index and outcomes in patients with heart failure [65], supporting the spleen as a potentially useful prognostic indicator in heart failure.

### Other functions of the spleen in the cardiovascular system

The most widely known function of the spleen is to provide macrophages that remove old or damaged erythrocytes and platelets from the circulation for subsequent degradation. Erythrocyte-derived hemoglobin is degraded into heme and globin, and iron molecules within the heme are subsequently recycled for the synthesis of de novo hemoglobin molecules. The spleen also stores erythrocytes. Hypoxia and exercise separately trigger spleen contraction, resulting in the release of stored erythrocytes [41]. A study in rats has shown that intermittent hypoxia results in a reversible increase in blood hemoglobin concentration during hypoxia via splenic contraction [66]. In a study of spleen volume and hemoglobin concentration in healthy individuals, the spleen contracted and mobilized stored erythrocytes at increased altitudes during rested conditions. Additionally, the spleen contracted further during exercise, increasing oxygen delivery to tissues during acute hypoxia [41]. From these observations, it was concluded that the attenuated hemoglobin response to exercise at high altitude was likely due to greater recruitment of the spleen reserve during rest and that maximal spleen contraction was reached during exercise [41]. Therefore, the spleen appears to play a role in regulating the circulating hemoglobin concentration in response to varying degrees of hypoxia in humans. In the context of chronic heart failure, patients with reduced ejection fraction experience dyspnea from fluid overload that may result in hypoxemia [67], which may in turn trigger the spleen to contract, mobilizing stored erythrocytes to regulate circulating hemoglobin concentration. Moreover, a recent study on the relationship between the spleen and exercise tolerance in patients with heart failure has shown that the link between splenic size and peak VO_2_ may be hemoglobin concentration [68]. Anemia and hemoglobin concentration [69–71] have previously been reported as prognostic factors in patients with heart failure. Thus, it is possible that spleen-mediated regulation of hemoglobin concentration is associated with heart failure prognosis.

## Clinical utility of the spleen as a prognostic biomarker in heart failure

In this paper, we have reviewed the available evidence on the cardiosplenic axis and the splenic manifestations that have been observed in heart failure to date. However, it is important that a consensus be reached on how these parameters can be measured clinically and how they may be useful to examine heart failure prognosis.

Of note, it is necessary to identify novel biomarkers to predict prognosis in patients with heart failure, for whom predicting risk is often challenging, and to provide a personalized strategy with precision medicine to improve outcomes.

The cardiosplenic axis represents a reservoir of immunological and hemodynamic processes that play a central pathophysiological role in heart failure. Therefore, it is our opinion that the spleen may be a useful tool to predict prognosis in patients with heart failure. At the macroscopic level, the size, volume, stiffness, and function of the spleen should be considered. At the microscopic level, there is a need to explore and develop molecular and cellular markers that are specific to the spleen, particularly those that can be evaluated by simple practical analyses, such as blood tests. Some of these factors are outlined in more detail below.

There is no established method to clinically assess spleen function in patients with heart failure at present; however, we speculate that certain immune cell types, such as proinflammatory C–C chemokine receptor 2 (CCR2)-positive macrophages, CD4 + T helper (Th; Th1, Th2, and Th17) cells, regulatory T cells, and CD8 + T cells, might be useful to predict heart failure prognosis. As explained by Prabhu [24], cardiac injury stimulates the death and substitution of cardiac-resident cells by infiltrating blood monocyte-derived macrophages, which express CCR2. Thus, CCR2-positive macrophages may be a useful marker of cardiac damage in heart failure. Sager et al. [53] have also shown that in chronic ischemic heart failure, the proportion of monocyte-derived macrophages increases. Expansion of CD4 + T helper cells and CD8 + T cells has also been observed in the spleen in heart failure [48, 72]. It is important to note that circulating immune cells are not necessarily specific to the heart and spleen; thus, suitable markers of the cardiosplenic axis that can be used to examine heart failure prognosis need to be identified.

Furthermore, when considering which factors might be most useful for prognostic prediction in patients with heart failure, Halade et al. [73] showed that leukocyte trafficking can be used to distinguish the status of inflammation in mice. Activated splenic immune cells modulate local tissue injury, cell death, and fibrosis in the heart, which influences cardiac remodeling, a topic previously reviewed by Prabhu [24]. Therefore, these cell types may be potentially useful for the assessment of inflammation and cardiac remodeling, and thus heart failure prognosis. It is also important to note that there are similarities and differences between different types of heart failure in terms of the correlative biomarkers. For example, Tromp et al. [74] showed that heart failure with preserved ejection fraction (HFpEF) or heart failure with mid-range ejection fraction is more closely related to inflammation than heart failure with reduced ejection fraction (HFrEF). In their study, pentraxin-3 (which regulates the inflammatory activity of macrophages [75]) was more associated with HFpEF. Another study [76] also showed that HFpEF is more associated with inflammation, while HFrEF is more associated with cardiac stretch. Given that HFpEF appears to be more closely related to inflammation, the spleen may play a greater role in this type of heart failure. However, further studies are needed to define the role of the spleen in HFpEF compared with HFrEF.

As described earlier, in patients with advanced heart failure, splenic size increases after LVAD implantation, and a significant negative correlation has been observed between splenic volume and systemic vascular resistance [21], which is often high in patients with heart failure. Thus, in terms of the clinical manifestations of heart failure that could be measured using the cardiosplenic axis, it is possible that splenic size and volume could inform on the functioning of the heart, such as its pumping efficiency or hemodynamics, and thus predict prognosis. However, when assessing the spleen in the context of heart failure, it would also be important to consider the association of the spleen with other organs and in different diseases. For example, the spleen has shown associations with the liver [77], obesity [78], and resting energy expenditure [78]. Thus, the spleen likely has a much wider range of functions than previously understood. When using the spleen as a prognostic tool in heart failure, such considerations would be particularly important in patients with co-existing diseases in which the spleen has demonstrated involvement. Further research is also needed to identify other functions of the spleen.

In terms of imaging approaches to assess spleen size and volume, computed tomography of the spleen could be used to monitor at-risk patients, although this method is relatively expensive and uses ionizing radiation. Ultrasound can also be used to measure spleen size, is more widely available than computed tomography, and does not use radiation, thus avoiding any potential damage from repeated measurements [79]. Doppler ultrasound may also be useful in measuring spleen function by evaluating spleen blood flow [80]. However, it is important to note that there is marked inter-user variability with ultrasound imaging. Moreover, shear-wave elastography to measure spleen stiffness could be used to quantify the severity of splenic congestion and is a viable method for predicting adverse events in patients with acute decompensated heart failure [81]. The use of ultrasound and shear-wave elastography to assess spleen size/volume/function and stiffness, respectively, as potential indicators of pumping efficiency and hemodynamics could provide a non-invasive alternative to invasive RHC, which would reduce the risk of complications.

## Future perspectives

A summary of the future perspectives and directions that should be taken to address the current knowledge gaps is shown in Table 1.

**Table 1:** Summary of future perspectives to advance knowledge on the prognostic role of the spleen in heart failure

**Perspective**	**Outcome**
**Step 1: Clarify animal observations in human studies**	A refined understanding of the relevance/importance of the cardiosplenic axis in humans
**Step 2: Clarify the role of the spleen in heart failure**	A deeper understanding of the interactions between the spleen and heart in heart failure to provide clarity on whether its pursuit as a prognostic indicator for heart failure is thoroughly justified This stage may help to identify reliable prognostic markers with potential clinical application
**Step 3: Determine whether splenic involvement is relevant to all types/stages of heart failure or whether it is specific to certain subpopulations**	Insight into whether the observed interactions (as described in Step 2) depend on the stage or etiology of heart failure and identification of subpopulations that might benefit from utilizing splenic measurements/biomarkers as prognostic indicators
**Step 4: Incorporate specific splenic measurements into established prognostic models for heart failure**	Evidence on how utilizing splenic measurements and/or biomarkers improves the efficacy of existing prognostic models for heart failure in the subpopulations identified in Step 3

First, it has been hypothesized that all organs possess endocrine function and may therefore contribute to whole-body homeostasis [82]. For example, the heart produces atrial natriuretic peptide and BNP; adipose tissues produce and secrete adipokines, such as adiponectin; and skeletal muscle produces and secretes myokines, such as myonectin, all of which may induce cardioprotective effects [83–86]. As such, further research is required to determine the role of the spleen within this hypothesized multi-organ endocrine system. In heart failure, the spleen may be instrumental in maintaining the functions of multiple organs, not just the heart. Currently, the hormones and endocrine substances that are specific to the spleen are unknown. It may be that the spleen secretes other factors in addition to macrophages and cytokines, such as hormones and chemical messengers, that may play a protective role in heart failure, which is an area worthy of further study.

Second, much of the existing evidence demonstrating the role of the spleen in heart failure is from animal studies; thus, further studies should be conducted in humans to clarify the relevance of the cardiosplenic axis and to identify potentially useful spleen-related prognostic predictors for heart failure.

Third, the physiological mechanisms of the role of the spleen in heart failure remain unclear, and the relationships between physiological indices and cellular and molecular markers specific to the spleen have not yet been clarified. Further knowledge in this area would be useful for the potential application of the spleen as a tool for prognostic prediction in heart failure.

Fourth, it has been emphasized that heart failure varies in etiology and pathology and can be highly variable; hence, splenic involvement may depend on the cause of heart failure. Therefore, its assessment may only be relevant for specific subpopulations of patients. If the cardiosplenic axis is demonstrated to play a definitive role in heart failure in humans, research to identify subpopulations of patients in whom the spleen may be a useful prognostic tool will be a valuable step toward precision medicine.

Finally, it has been established that prognostic models for heart failure are more effective than individual clinical variables alone [9]. Thus, if the spleen is confirmed as a useful tool for prognostic prediction in heart failure, future studies should investigate the most effective way to incorporate spleen assessment into existing prognostic models.

## Conclusions

In this study, we reviewed evidence of the interplay between the heart and spleen, termed the cardiosplenic axis, in cardiovascular disease and heart failure. The spleen forms a major part of the immune system, and evidence suggests a role for the spleen in immune cell mobilization as a mediator of inflammation and cardiac remodeling. Other evidence suggests a relationship between splenic size and systemic blood volume regulation and hemodynamics. Most studies have been conducted in animals; thus, future studies in humans should aid our understanding of the importance of the cardiosplenic axis and drawing of definitive links between specific splenic measurements and different heart failure manifestations, including hemodynamic changes, inflammation, fibrosis, and remodeling. It should also be determined whether splenic measurements have variable importance between different heart failure classes and etiologies. These contributions will represent a step forward in understanding the usefulness of the spleen as a prognostic predictor in heart failure.
